# Supplementary material for: Sustainability of a Multi-Component Education Program (ABC of Healthy Eating) after Three Months and Nine Months: The Socioeconomic Context in Improving Nutrition Knowledge in Polish Teenagers
Source: Nutrients. 2021 May 14;13(5):1661. doi: 10.3390/nu13051661 (PMC8153617; doi:10.3390/nu13051661)
Supplement: Supplementary file 1 [file nutrients-13-01661-s001.zip › nutrients-1195818-supplementary.pdf]

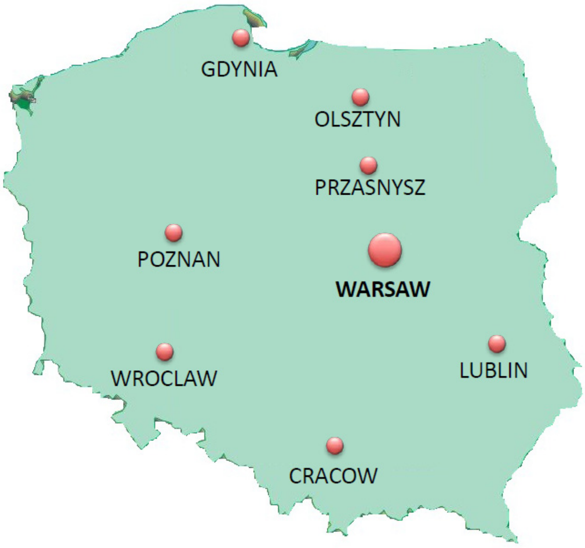

Figure S1. Location of schools included in the study (up to 50 km from the academic centres) and academic centres involved in the study (in brackets): Warsaw (Warsaw University of Life Sciences, WULS-SGGW), Przasnysz (Warsaw University of Life Sciences, WULS-SGGW); Gdynia (Gdynia Maritime University); Cracow (University of Agriculture in Krakow); Lublin (University of Life Sciences in Lublin); Poznan (University of Life Sciences in Poznan); Olsztyn (University of Warmia and Mazury in Olsztyn); Wroclaw (Wroclaw University of Environmental and Life Sciences).

Table S1. Topics and details of the education program

**Nutrition topic**	
Goal	Shaping pro-healthy dietary habits.
Scope	Nutrients important in the diet of young people. Health consequences of uncontrolled consumption of energy drinks and dietary supplements. A 'Good snack' as an alternative to chips, sticks and sweets. A Pyramid of Healthy Nutrition and Physical Activity and dietary guidelines for teenagers.
Methods	Talk; discussion; workshops.
Activities	Participants propose three various 'healthy' breakfasts to take to school by selecting foods from levels of the Pyramid of Healthy Nutrition and Physical Activity; Participants prepare an 'ideal sandwich'.
Tools	Brochure; puzzles; crosswords; website.
**Dietary topic**	
Goal	Supporting well-being, physical and intellectual development through a healthy lifestyle.
Scope	General recommendations for a healthy lifestyle (healthy eating and physical activity) at school age. The influence of nutrition and physical activity on physical and intellectual development and healthy well-being. The Student Menu – the brain cannot live only on chocolate – a discussion on the most important nutrients in the diet of young people.
Methods	Talk; discussion; workshops.
Activities	Using a pedometer to measure the number of calories 'burned' during the various activities. Determination of time needed to 'burn' the calories coming from the selected food products. 'Nutrition detective' – measuring the amounts of oil, sugar and salt corresponding to fat, sucrose and salt content in various foods (three sets).
Tools	Brochure; diverse food products; puzzles; crosswords; pedometer; website.
**Sensory-consumer topic**	
Goal	The world of senses. How to read the food labels? What is important when choosing food?
Scope	Recognition of the basic flavours in aqueous solutions. Discovering the different taste sensations in selected foods. 'What kind of a consumer am I' – what do I like and why? Qualification of the consumer's personality.
Methods	Talk; discussion; workshops.
Activities	Use of sensory memory to identify eight coded odour samples of natural spices, vegetables and fruits. Recognition of selected foods with masked/closed eyes, based on sensory perceptions in the mouth. Preparation of colourful, tasty and healthy snacks from provided foods according to one's own ideas.
Tools	Brochure; recipes of 'healthy' snacks; website.
**Hygiene topic**	

Goal	Food safety. Hygiene during the preparation and consumption of meals.
Scope	The world of microorganisms, pathogens and probiotics. The rules of proper food storage and hygiene during meal preparation and consumption.
Methods	Talk; discussion; workshops.
Activities	Microscopic observation of selected microorganisms – Lactic acid bacteria (Lactobacillus) as an example of a microorganism with healthy properties, E. coli (Escherichia coli) as an example of a pathogen. Mapping the observed microorganisms' cells in the prepared templates. Practicing proper hand washing according to instructions. Checking hand cleanness with a test indicator, before and after hand washing.
Tools	Brochure; microscope; test indicator of hand washing; puzzles; crosswords; website.
**Culinary topic**	
Goal	How to prepare healthy, cheap and tasty meals?
Scope	The impact of culinary processes on sensory quality, nutritional value and food safety. The phenomenon of enzymatic browning of fruit and vegetables and ways to prevent this process.
Methods	Talk; discussion; workshops.
Activities	Culinary experiments – preventing the darkening of fruits and vegetables. Checking the impact of storage temperature on the quality of frozen foods, *e.g*. vegetables. Preparing low-budget healthy meals and low-sweetened beverages.
Tools	A 'healthy meals' recipe book; brochure; website.

Table S2. Questions and correct answers (scored with 1 point) regarding the nutrition knowledge score (NKS).

Question	Correct answer (scored with 1 point)
Q1: The key of healthy eating is:	Three answers are correct: (a) to eat many different kinds of foods; (b) to eat more of some products than others; (c) to eat moderate or small amounts of some products.
Q2: How much of milk and milk beverages *e.g.* kefir, yoghurt should you eat during a day?	Two glasses.
Q3: How much fruits and vegetables should you eat?	Five or more fruits and vegetables a day.
Q4: 'Good' microorganisms are such microorganisms which:	Three answers are correct: (a) cause food fermentation; (b) cause bread rising; (c) have positive influence on human health.
Q5: Fast foods contain much of:	Fat and salt.
Q6: Which set of products contains much fibre?	Whole-meal bread, apple, bean.
Q7: Main sources of calcium in diet are:	Dairy products.
Q8: Which breakfast set contains less fat?	Corn flakes with full fat milk.
Q9: Main function of protein in the body is:	Regulation and structure.
Q10: Alkalizing products are:	Fruits, vegetables and potatoes.
Q11: Which information on the food label is most important for every consumer's food safety?	Shelf life.
Q12: Vegan diet means that one is eliminating from diet:	Meat, dairy and eggs.
Q13: Amount of human energy requirement depends on:	Age, sex and physical activity.
Q14: BMI index is a ratio of:	Body weight and height.
Q15: To be active means:	All above answers.
Q16: Which way of cooking is the best for vitamin C retention in potatoes?	Steam cooking.
Q17: Where one should keep an open container of juice:	In refrigerator.
Q18: Energy drinks are safe for adolescences:	Should not be consumed before age of 16.

Table S3. Questions and response categories (with scoring) regarding the Family Affluence Scale (FAS) [35].

Question	Response categories with scoring
1. Does your family own a car, van or truck?	no (0 points); yes, one (1 point); yes, two or more (2 points)
2. During the past year, how many times did you travel away on holiday with your family?	not at all (0 points); once (1 point); twice (2 points); more than twice (2 points)
3. Do you have your own bedroom for yourself?	no (0 points); yes (1 point)
4. How many computers, laptops or tablets does your family own?	none (0 points); one (1 point); two (2 points); more than two (2 points)
